# Cloning and Functional Analysis of Rat *Tweety-Homolog 1* Gene Promoter

**DOI:** 10.1007/s11064-021-03374-2

**Published:** 2021-06-26

**Authors:** Malgorzata Gorniak-Walas, Karolina Nizinska, Katarzyna Lukasiuk

**Affiliations:** Laboratory of Epileptogenesis, Nencki Institute of Experimental Biology, Polish Academy of Sciences, 3 Pasteur Street, 02-093 Warsaw, Poland

**Keywords:** Dual luciferase assay, Promoter, Transcription factors, Ttyh1

## Abstract

*Tweety-homolog 1* protein (*Ttyh1*) is abundantly expressed in neurons in the healthy brain, and its expression is induced under pathological conditions. In hippocampal neurons in vitro, Ttyh1 was implicated in the regulation of primary neuron morphology. However, the mechanisms that underlie transcriptional regulation of the *Ttyh1* gene in neurons remain elusive. The present study sought to identify the promoter of the *Ttyh1* gene and functionally characterize *cis*-regulatory elements that are potentially involved in the transcriptional regulation of *Ttyh1* expression in rat dissociated hippocampal neurons in vitro. We cloned a 592 bp rat *Ttyh1* promoter sequence and designed deletion constructs of the transcription factors specificity protein 1 (Sp1), E2F transcription factor 3 (E2f3), and achaete-scute homolog 1 (Ascl1) that were fused upstream of a luciferase reporter gene in pGL4.10[*luc2*]. The luciferase reporter gene assay showed the possible involvement of Ascl1, Sp1, and responsive *cis*-regulatory elements in *Ttyh1* expression. These findings provide novel information about *Ttyh1* gene regulation in neurons.

## Introduction

The *Tweety-homolog1* (*Ttyh1*) gene encodes a transmembrane protein that putatively functions as a chloride channel [1–4]. *TTYH1* is expressed in embryonic stem cells both during the early stages of brain development and in the adult brain [5]. Recently, *TTYH1* has been discovered as one of the specific progenitor genes in human developing hypothalamus [6]. Ttyh1 is highly expressed in neurons in the healthy rat brain and dissociated hippocampal neurons, regardless of the age of the culture [7–12]. Ttyh1 was detected in neuropils, neuronal somata [8, 12], the presynaptic active zone of the rat brain [9], and invaginations of dendritic spines in vitro [12].

The importance of Ttyh1 in neural function has been underscored by its recently documented involvement in the regulation of neural morphology in vitro and aberrant neuronal structural plasticity in vivo [12]. Elevations of Ttyh1 expression were detected in the molecular layer of the dentate gyrus during epileptogenesis [11–13]. Our recent study revealed that Ttyh1 participates in shaping dendritic tree and dendritic spines morphology in hippocampal slices in vitro (Gorniak-Walas, submitted).

*Ttyh1* mRNA is highly expressed in neurons in vitro and in vivo [7–12], but current knowledge of the transcriptional regulation of *Ttyh1* in neurons is still lacking. Transcriptional regulation is a highly coordinated process and required for temporal- and tissue-specific gene expression. Therefore, the ability to identify promoter sequences and predict specific transcription factor binding sites is integral to unraveling the mechanism of *Ttyh1* gene regulation.

## Results

### In silico Characterization of the *Ttyh1* Gene Promoter

**Fig. 1:**
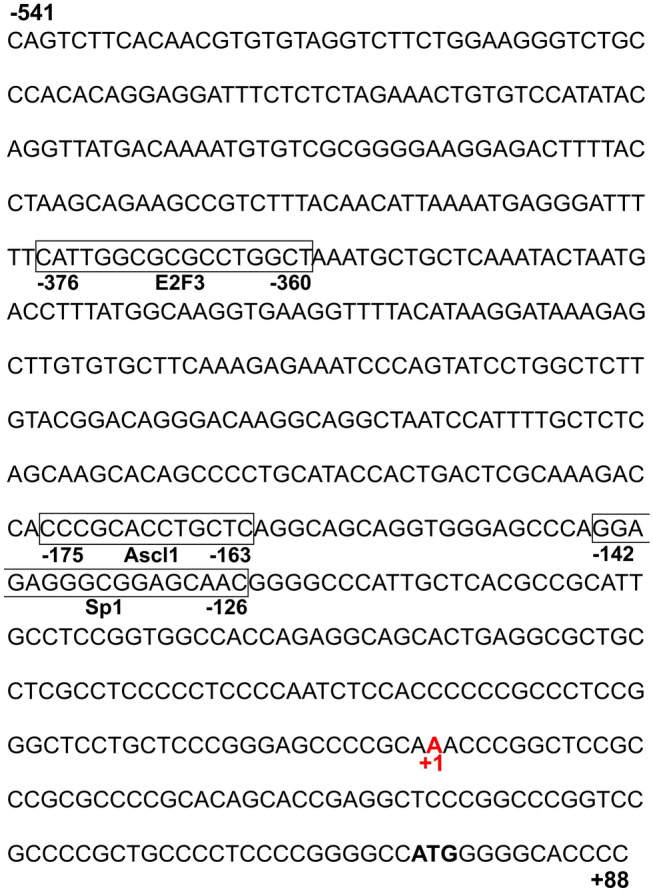
The rat *Ttyh1* promoter sequence obtained from MatInspector software. Putative binding sites for E2f3, Ascl1, and Sp1 are in boxes. The ATG initiation codon is in bold, and the TSS is highlighted in red (marked as + 1)

**Fig. 2:**
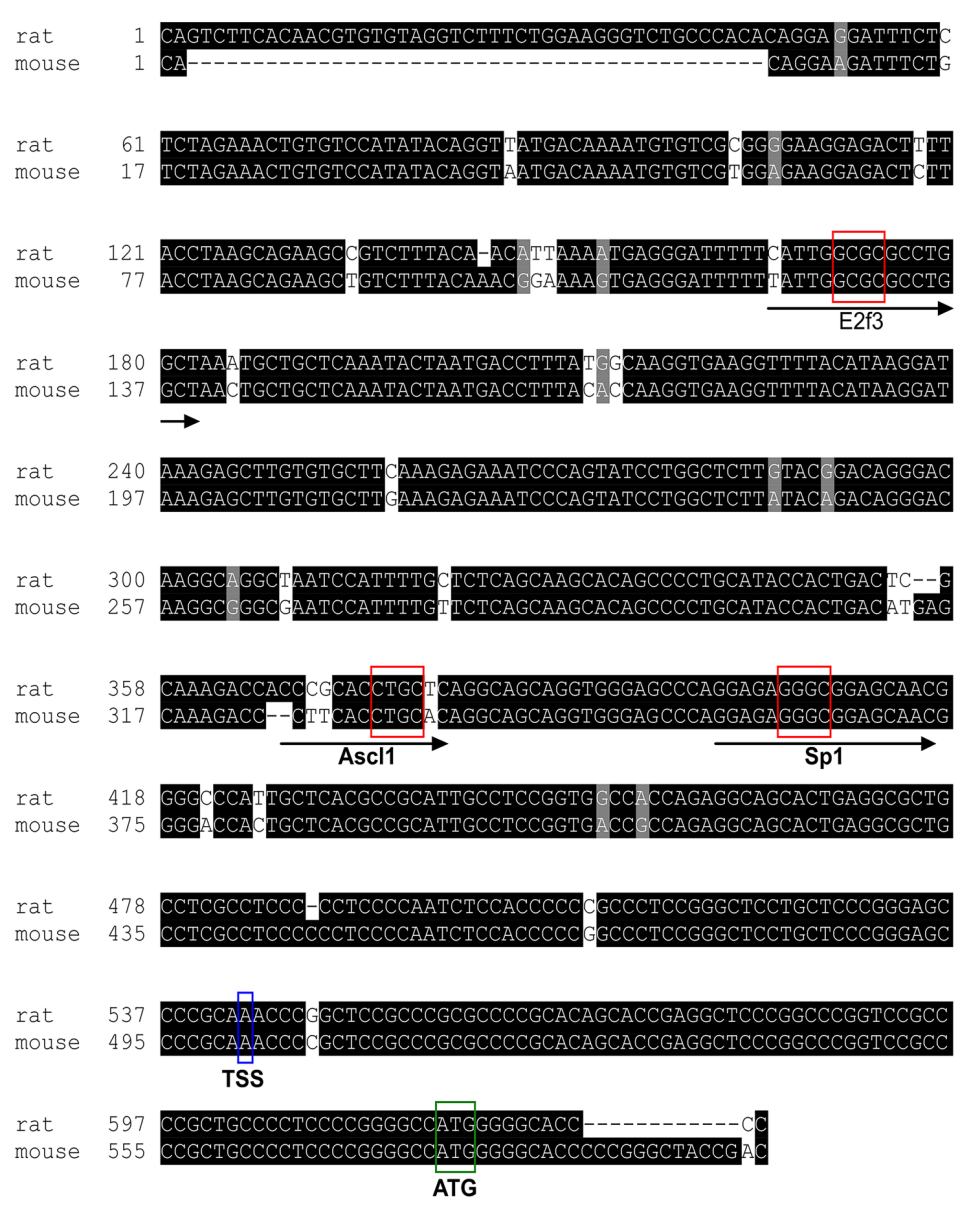
Amino acid alignment of the rat (*R. norvegicus*) and mouse (*M. mulsculus*) *Ttyh1* gene promoter (NM_001106225 and NM_021324, respectively). Black and grey boxes indicate identical and similar nucleotides. Putative binding sites for E2f3, Ascl1, and Sp1 are indicated by arrows. The core sequences are in red boxes. The TSS and the ATG initiation codon are highlighted by blue and green boxes, respectively. The sequence alignment was carried out using T-Coffee, the alignment was formatted using BoxShade

To gain insights into the transcription regulation of neuronal *Ttyh1* expression, we analyzed the *Ttyh1* promoter sequence in search of putative transcription factor binding sites. Using MatInspector software, we isolated a 630 bp fragment that contained the rat *Ttyh1* promoter (Fig. 1). To identify the transcription start site (TSS) in the rat *Ttyh1* promoter, the sequences of the mouse *Ttyh1* promoter were retrieved from the Eukaryotic Promoter Database. Promoter sequences of rat *Ttyh1* showed 93% sequence similarity to the mouse *Ttyh1* promoter (Fig. 2). We found that the TSS was located 75 nt upstream of the ATG initiation codon (marked as + 1 in Figs. 1, 2).

### Identification of Putative Binding Sites in the Rat *Ttyh1* Gene Promoter

To identify potential *cis*-regulatory elements within the *Ttyh1* promoter, we used MatInspector software. Although the *Ttyh1* promoter included a number of *cis*-regulatory elements, we selected high-score potential binding sites for Sp1, Ascl1, and E2f3 upstream of TSS (−142 to −126 bp, 175 to −163 bp, and −376 to −360 bp, respectively; Figs. 1, 2, Table 1).

**Table 1:** Binding sites for specific regulatory proteins with the highest degree of homology between species (MatInspector software)

No	Matrix similarity [0.9–1]	Core sequence similarity [0.9–1]	Potential transcription factor for binding site	Tissue	Transcription factor function
1	1	1	ASCL1	Neurons, glia	Gene expression regulation during neurogenesis
2	0.931	1	E2F3	Ubiquitous	Cell cycle regulation
3	0.928	1	SP1	Ubiquitous	Gene expression regulation in brain pathology

### Functional Characterization of Regulatory Elements in the Rat *Ttyh1* Gene Promoter

To interrogate the role of *cis*-regulatory elements in *Ttyh1* expression, we cloned a 592 bp rat *Ttyh1* promoter sequence (−541 to + 51 bp), designed deletion constructs for Sp1, E2f3, and Ascl1 that were fused to a luciferase reporter gene in pGL4.10[*luc2*], and performed a dual luciferase reporter assay in dissociated hippocampal neurons in vitro. This method allows identification of nucleotide sequences in the promoter region responsible for the regulation of gene expression [14].

**Fig. 3:**
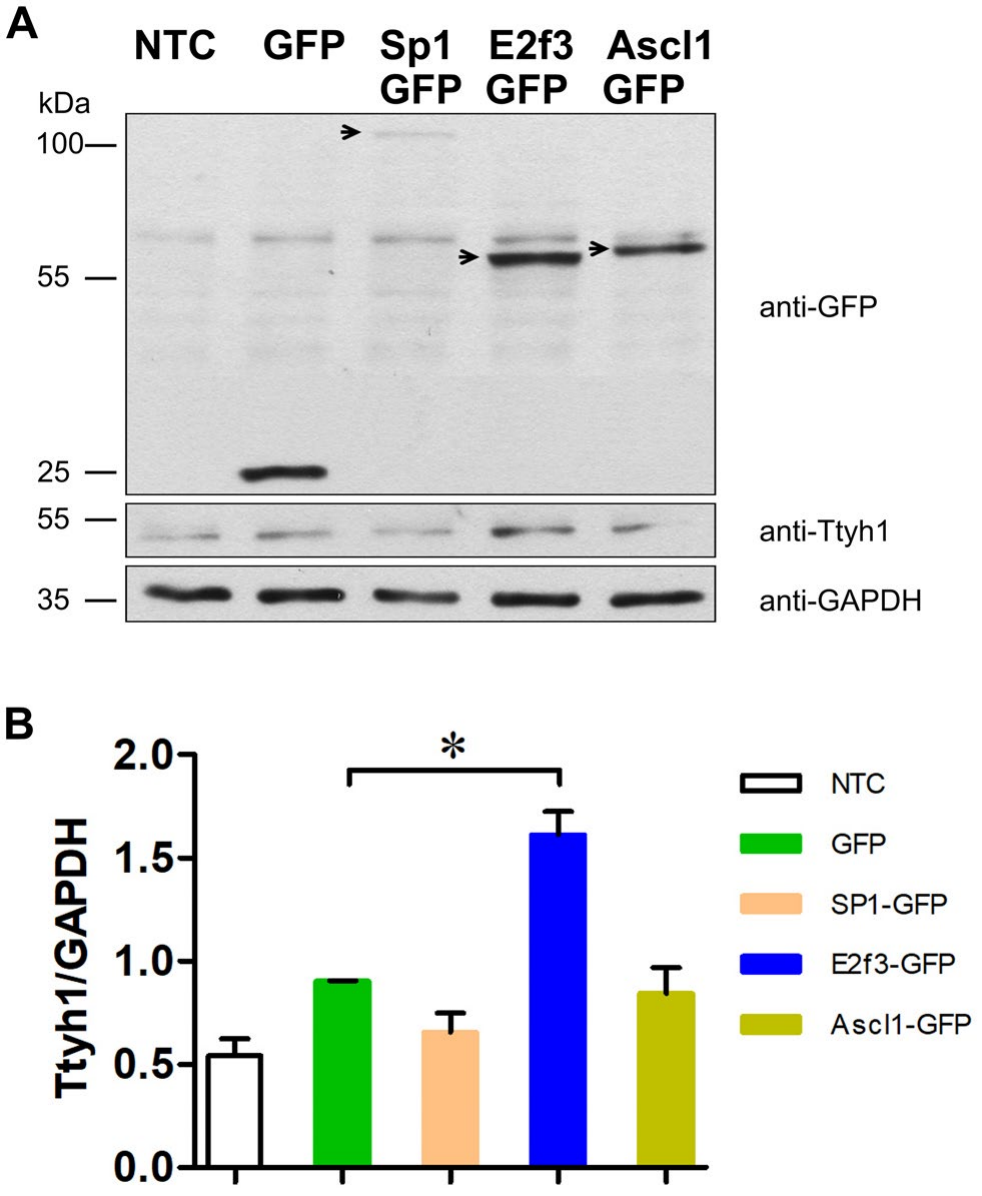
Expression levels of Sp1-GFP, E2f3-GFP, Ascl1-GFP, and Ttyh1 proteins following transfection with respective vectors in hippocampal neurons in vitro*.* **A** Western blot analysis of the expression level of Sp1-GFP, E2f3-GFP, Ascl1-GFP, and Ttyh1 proteins. The arrows show the overexpressed Sp1-GFP, E2f3-GFP, and Ascl1-GFP, respectively. GAPDH was used as a loading control. **B** The intensity of Ttyh1 expression was normalized to GAPDH

The coding sequences of E2f3, Ascl1, and SP1 were amplified by PCR and cloned into an N1-GFP plasmid. The expression of E2f3-GFP, Ascl1-GFP, and Sp1-GFP, was confirmed by Western blot (Fig. 3a, b). The transfection efficiency achieved using Lipofectamine2000 was lower for N1-EGFP plasmid contained the coding sequence of Sp1 (2361 bp) compared to plasmid contained the coding sequence of E2f3 (738 bp), and Ascl1 (702 bp). As the size of the plasmid can influence the transfection efficiency, transfection of hippocampal neurons with N1-Sp1-GFP (the total size of the plasmid: 7061 bp) could result in lower DNA uptake by the cells compared to N1-E2F3-GFP (the total size of the plasmid: 5438 bp) and N1-Ascl1-GFP (the total size of the plasmid: 5402 bp). Interestingly, we detected a 2.8-fold increase in Ttyh1 expression in E2f3-GFP-expressing neurons compared with control neurons (Fig. 3a, b).

**Fig. 4:**
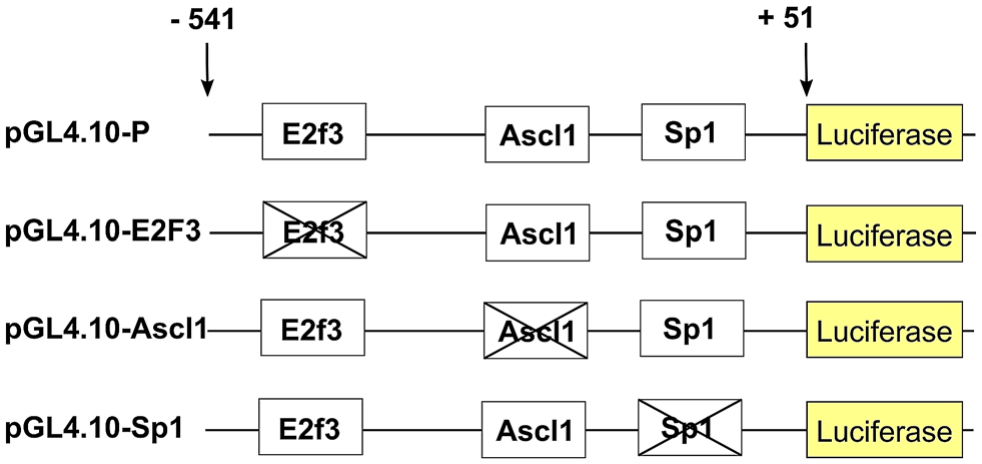
The luciferase constructs used in the experiments. Schematic representation of *Ttyh1* promoter construct (pGL4.10-P), and the deletion constructs of the E2F3 (pGL4.10-E2f3), Ascl1 (pGL4.10-Ascl1), and Sp1 (pGL4.10-Sp1) used in the luciferase assay

Deletion constructs were devoid of predicted binding sites for E2f3 (−376 to −360 bp), Ascl1 (−175 to −163 bp), and Sp1 (−142 to −126 bp), that were linked upstream of the luciferase reporter gene in pGL4.10[*luc2*] (Fig. 4). The promoter deletion constructs were generated using site-directed mutagenesis by overlap extension based on polymerase chain reaction (PCR).

Dissociated hippocampal neurons were transfected with the N1-GFP empty plasmid, or N1-GFP plasmid that contained coding sequences of E2f3, Ascl1, and Sp1 (N1-E2f3-GFP, N1-Ascl1-GFP, and N1-Sp1-GFP, respectively), and the pGL4.74[*hRluc*/TK] plasmid as an expression control. These plasmids were co-transfected with the pGL4.10[*luc2*] empty plasmid or pGL4.10[*luc2*] plasmid that encoded the *Ttyh1* promoter (pGL4.10-P), and luciferase activity was monitored.

The analysis of promoter activity revealed that the *Ttyh1* promoter (pGL4.10-P) exhibited high transcriptional activity, ensuring a strong level of luciferase gene expression.

**Fig. 5:**
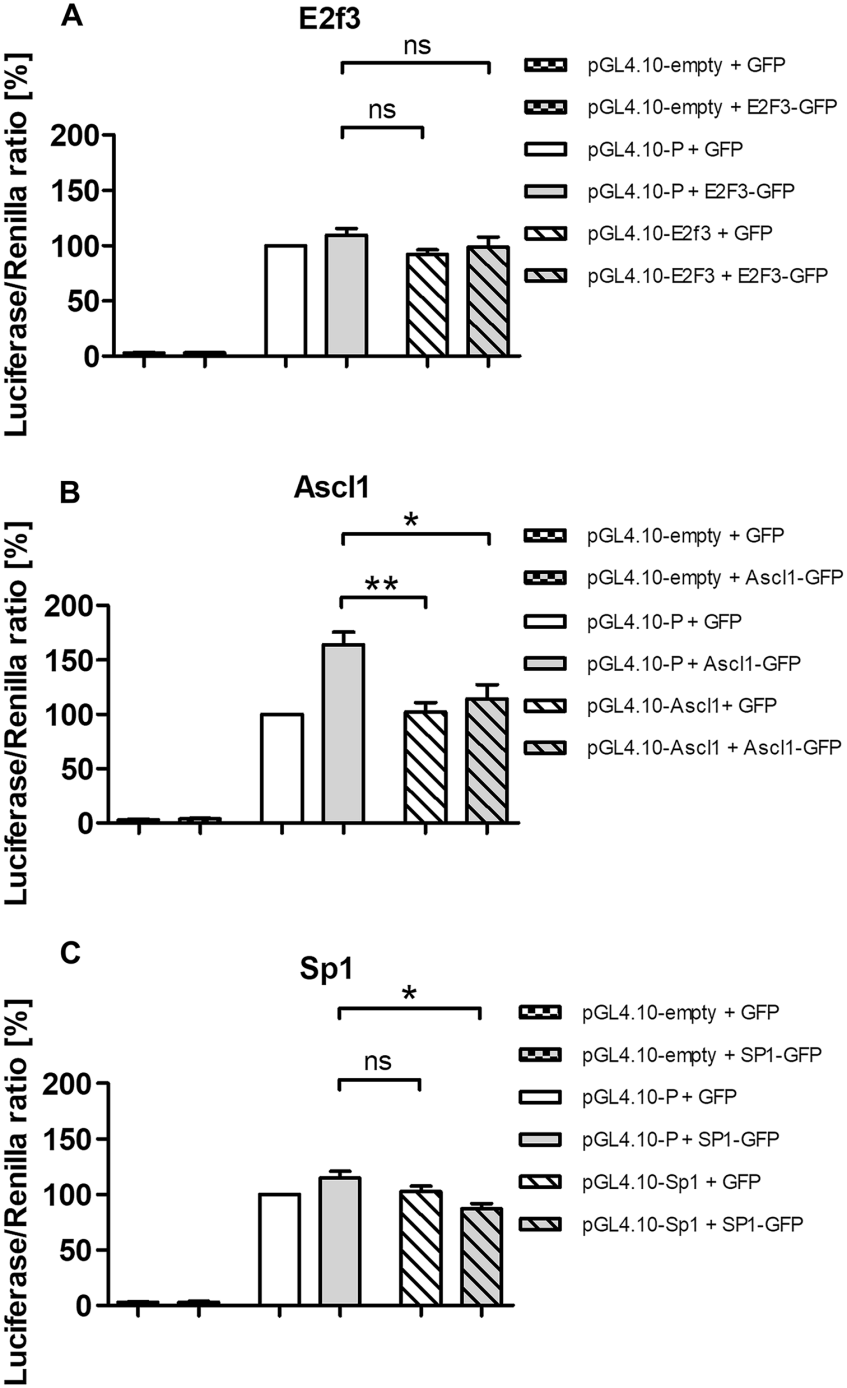
Transcriptional regulation of *Ttyh1* promoter in hippocampal neurons in vitro. Luciferase reporter gene assays were used to determine *Ttyh1* promoter activity in hippocampal neurons in vitro for the transcription factors E2f3 (**A**), Ascl1 (**B**), and Sp1 (**C**). The data are expressed as mean ± SEM. The data were collected from four biological replicates. Three technical replicates were performed for each sample. ^*^p < 0.05, ^**^p < 0.01 (two-tailed Student’s *t*-test)

This basal *Ttyh1* promoter activity was 25-fold higher than the pGL4.10[*luc2*] empty plasmid and set to 100% for further comparisons (Fig. 5).

To investigate the functional role of E2f3, Ascl1, and Sp1 in the transcriptional regulation of *Ttyh1* gene expression, we examined the effects of the overexpression of individual transcription factors on activity of the *Ttyh1* gene promoter. As shown in Fig. 5a and c, we did not observe significant differences in promoter activity between E2f3-GFP- or Sp1-GFP-expressing neurons and control neurons that expressed GFP only. However, the *Ttyh1* promoter exhibited 1.6-fold higher activity following Ascl1-GFP overexpression compared with basal *Ttyh1* activity (Fig. 5b).

To further examine the functional role of predicted binding sites in *Ttyh1* gene expression, we designed promoter deletion constructs for E2f3 (pGL4.10-E2f3), Ascl1 (pGL4.10-Ascl1), and Sp1 (pGL4.10-Sp1) predicted binding sites. As shown in Fig. 5, the promoter deletion constructs had basal activity that was similar to the full-length *Ttyh1* promoter (pGL4.10-P). The activity of promoter deletion construct for Ascl1 (pGL4.10-Ascl1) was significantly lower in GFP-expressing neurons compared to the activity of the full-length *Ttyh1* promoter (pGL4.10-P) in Ascl1-GFP-overexpressing neurons (*p* < 0.01). Interestingly, the deletion construct for Ascl1 (pGL4.10-Ascl1) significantly abolished *Ttyh1* promoter activity in Ascl1-GFP-overexpressing neurons (*p* < 0.05) (Fig. 5b). The *Ttyh1* promoter activity of deletion construct for Sp1 (pGL4.10-Sp1) was significantly decreased in Sp1-GFP-overexpressing neurons (*p* < 0.05) (Fig. 5c).

These data suggest that the Sp1 and Ascl1 transcription factors may be involved in the transcriptional regulation of *Ttyh1* expression in neurons.

## Discussion

*TTYH1* is primary expressed in embryonic stem cells and continues to be expressed at later stages of brain development [5]. *Ttyh1* expression is induced under pathological conditions, such as epilepsy [11–13], triple-negative breast cancer [15], glial tumors [16, 17], and in activated astrocytes in the epileptic brain [18]. To date, the mechanisms that underlie transcriptional regulation of the *Ttyh1* gene remain elusive.

We generated a promoter of the rat *Ttyh1* gene and identified *cis*-regulatory elements presumably involved in the transcriptional regulation of *Ttyh1* in rat dissociated hippocampal neurons in vitro. We cloned a 592 bp rat *Ttyh1* promoter sequence that was fused upstream of a luciferase reporter gene in pGL4.10[*luc2*] and designed promoter deletion constructs for Sp1 (−142 to −126 bp), Ascl1 (−175 to −163 bp), and E2f3 (−375 to −360 bp) predicted binding sites. Our findings provide evidence of the involvement of Sp1 and Ascl1 in *Ttyh1* gene regulation in neurons.

Aachaete-scute like 1 (Ascl1) belongs to the basic-helix-loop-helix family and is expressed in the nervous system [19–22]. Ascl1 regulates gene expression during neurogenesis and controls cell-fate determination in neurons and glia [23–26]. Ascl1 has also been implicated in Parkinson’s disease and various cancers (e.g., glioma and neuroblastoma) [27, 28]. For example, high Ascl1 expression in glioma promotes neuronal differentiation and prevents tumor growth [29]. Ttyh1 regulates embryonic neural stem cell properties by positively regulating the Notch pathway [30, 31]. Evidence from human studies indicates that *Ttyh1* is expressed in embryonic stem cells and neural structures and continues to be expressed at later stages of brain development [5]. These findings are consistent with our previous studies, in which Ttyh1 was expressed at high levels in rat hippocampal neurons in vitro, regardless of the age of the culture [12]. The present study showed a 1.6-fold increase in *Ttyh1* promoter activity in Ascl1-GFP-overexpressing neurons compared with control neurons and significantly abolished activity of the deletion construct for Ascl1 (*p* < 0.05) compared with the full-length *Ttyh1* promoter following Ascl1-GFP overexpression. Given the developmental function of Ascl1 in the central nervous system and high expression of *Ttyh1* in embryonic stem cells and during brain development [5], Ascl1 appears to govern *Ttyh1* gene expression during embryonic development.

Transcription factor Sp1 is widely expressed and regulates the expression of numerous genes that are involved in various processes, such as cell growth, metabolism, differentiation, and the immune response [32]. High SP1 expression was detected in the hippocampus after kainic acid administration in rats [33] and in the postmortem hippocampus in chronic schizophrenia patients [34]. Sp1 has been also implicated in tumorigenesis [35]. Previous studies implicated *Ttyh1* in pathological conditions, such as epilepsy [11, 13] and glial tumor progression [16, 17]. In the present study, we observed a significant decrease in activity of the deletion construct for Sp1 compared with *Ttyh1* promoter activity in Sp1-GFP-overexpressing neurons. Thus, Sp1 appears to be involved in *Ttyh1* gene regulation in disease-related processes.

Transcription factor E2f3 plays an important role in regulating the cell cycle [36], and its dysregulation has been implicated in human cancers [37–39]. Previous studies indicated that miRNAs regulate the expression of E2f3 in various types of cancers. For example, *E2f3* expression is posttranscriptionally regulated by miR-128 in glioma [40]. Under these conditions, miR-128 is downregulated, and E2F3 is highly expressed. Interestingly, miR-128 was significantly downregulated in the dentate gyrus and CA1 layer of the hippocampus in epileptic rats [41, 42]. An increase in *Ttyh1* expression has been implicated in glial tumors [16, 17] and epilepsy [11, 13]. Therefore, we speculate that miR-128 may target E2f3 and regulate the expression of *Ttyh1* under pathological conditions. Although the luciferase reporter gene assay in the present study did not reveal an impact of E2f3 on activity of the *Ttyh1* promoter in hippocampal neurons in vitro, we observed a 2.8-fold increase in Ttyh1 expression in E2f3-GFP-expressing neurons compared with control neurons. Therefore, we cannot exclude the possibility that E2f3 interacts with another *cis*-regulatory sequence within the *Ttyh1* promoter and drives the expression of *Ttyh1* under pathological conditions.

The relevance of Ttyh1 to neuronal function is underscored by its high expression in the healthy brain and the induction of its expression in disease-related processes. The present study suggests that Ascl1 and Sp1 play a potential role in the modulation of *Ttyh1* expression under physiological conditions or in brain pathology. The regulation of *Ttyh1* expression appears to play a prominent role in proper brain development and function. Further studies should explore the precise mechanism of the interaction between Ascl1 and Sp1 and responsive *cis*-regulatory elements within the *Ttyh1* promoter.

## Materials and Methods

### Sequence Analysis of the *Ttyh1* Gene Promoter

To identify the promoter sequence of the rat *Ttyh1* gene and check for potential binding sites, we used MatInspector software (MatInspector Release Professional 8.2, December 2014) [43]. For functional analysis, we selected transcription factor binding sites with a high degree of homology between species.

### Cloning of the Rat *Ttyh1* Gene Promoter

The rat genomic DNA was extracted from the tail using Genomic Mini (catalog no. 116-50, A&A Biotechnology, Gdynia, Poland). The rat *Ttyh1* gene promoter was amplified from extracted genomic DNA using Phusion High-Fidelity DNA Polymerase (catalog no. F530L, Thermo Fisher Scientific, Waltham, MA, USA; forward primer, 5’-CAGTCTTCACAACGTGTGTAGGT-3’; reverse primer, 5’-TGCTGGTACTCTTGGTCGCG-3’). A 592 bp promoter sequence of the rat *Ttyh1* gene upstream of the TSS was obtained by PCR using 5’-TGCA*GCTAGC*CAGTCTTCACAACGTGTGT-3’ (forward) and 5’-TAT*AAGCTT*GACCGGGCCGGGAGC-3’ (reverse) primers that carried *Nhe*I and *Hind*III restriction sites, respectively. To design the *Ttyh1* promoter construct (pGL4.10-K), the PCR product was cloned 5’-upstream of a luciferase reporter gene into pGL4.10[*luc2*] (catalog no. E6651, Promega, Madison, WI, USA) by restriction enzyme digestion using *NheI* (catalog no. R3131S, New England BioLabs, Ipswich MA, USA), *HindIII* (catalog no. R3104S, New England BioLabs, Ipswich MA, USA), and the Quick Ligation Kit (catalog no. M2200S, New England BioLabs, Ipswich MA, USA). Promoter deletion constructs that were devoid of the predicted binding site for Sp1 (−142 to −126 bp; pGL4.10-Sp1), E2f3 (−376 to −360 bp; pGL4.10-E2f3), and Ascl1 (−175 to −163 bp; pGL4.10-Ascl1) were designed using site-directed mutagenesis by overlap extension and PCR. Briefly, the promoter construct (pGL4.10-P) was amplified in two separate PCRs using F and R2 primers and F2 and R primers, with R2 and F2 overlapping primers (15 nt 3’-overhangs) upstream (R2) or downstream (F2) of the sequence that was deleted. The two PCR products, F-R2 and F2-R, were amplified in one reaction using forward and reverse primers. The mutated PCR product was introduced by restriction enzyme digestion (*Nh*eI, *Hind*III) and ligation (Quick Ligation Kit, catalog no. M2200S, New England BioLabs, Ipswich MA, USA). The following primers were used for the *Ttyh1* promoter deletion constructs: F_Sp1 (5’-TGCA*GCTAGC*CAGTCTTCACAACGTGTGT-3’), R_Sp1 (5’-TAT*AAGCTT*GACCGGGCCGGGAGC-3’), R2_Sp1 (5’-AATGGGCCCCTGGGCTCCCACCT-3’), F2_Sp1 (5’-AGGTGGGAGCCCAGGGGCCCAT-3’), F_E2f3 (5’-TAT*GCTAGC*CAGTCTTCACAACGT-3’), R_E2f3 (5’-TAT*AAGCTT*GACCGGGCCGG-3’), R2 (5’-ATTTGAGCAGCATTTAAAAATCCCTCATTT-3’), F2_E2f3 (5’-AAATGAGGGATTTTTAAATGCTGCTCAAAT-3’), F_Ascl1 (5’-TGCA*GCTAGC*CAGTCTTCACAACGTGTGT-3’), R_Ascl1 (5’-TAT*AAGCTT*GACCGGGCCGGGAGC-3’), R2_Ascl1 (5’-CACCTGCTGCCTTGGTCTTTGCGAG-3’), and F2_Ascl1 (5’-CTCGCAAAGACCAAGGCAGCAGGTG-3’). The sequences of all of the constructs were verified by sequence analysis.

### Cloning of Transcription Factor Coding Sequences

The N1-EGFP plasmids that contained the coding sequences of Sp1, E2f3, and Ascl1 were obtained by PCR using Phusion High-Fidelity DNA Polymerase (catalog no. F530S, Thermo Fisher Scientific, Waltham*,* MA*,* USA), a forward primer that carried the *EcoR*I restriction site, a reverse primer that carried the *Age*I restriction site, and pCMV6-Sp1 (NM_012655, catalog no. RN213136, OriGene, Rockville, MA, USA), pCMV6-E2f3 (NM_001137626, catalog no. RN207430, OriGene Rockville, MA, USA), or pCMV-Ascl16 (NM_022384, catalog no. RN215503, OriGene Rockville, MA, USA) as a template. The PCR product was introduced into the N1-EGFP plasmid by restriction enzyme digestion (*Age*I, catalog no. ER1462, Thermo Fisher Scientific, Waltham*,* MA*,* USA; *EcoR*I, catalog no. ER0271, Thermo Fisher Scientific, Waltham*,* MA*,* USA) and ligation (Quick Ligase, catalog no. M2200S, New England BioLabs, Ipswich MA, USA). The following primers were used to generate the expression constructs: F_Sp1 (5’-GCTAT*GAATTC*GCCATGAGCGACCA-3’), R_Sp1 (5’-ACTA*ACCGGT*GAGAAACCATTGCCAC-3’), F_E2f3 (5’-GCTAT*GAATTC*GCCATGAGAAAGGG-3’), R_E2f3 (5’-AGCT*ACCGGT*GAATTTTTCGAATATCTTG-3’), F_Ascl1 (5’-GAT*GAATTC*ATTATGGAGAGCTCT-3’), and R_Ascl1 (5’-AGCT*ACCGGT*GAGAACCAGT-3’).

### Dissociated Hippocampal Neurons In Vitro

Primary cultures of hippocampal neurons were prepared under sterile conditions from embryonic day 18 Wistar rat embryos (Animal House, Nencki Institute of Experimental Biology, Warsaw, Poland) according to a previously described procedure [12, 44]. All of the procedures were performed in accordance with the Animal Protection Act of Poland (Directive 2010/63/EU). No ethical approval is required under Polish law for tissue collection. Pregnant rats were anesthetized with 4% isoflurane, followed by swift decapitation with guillotine. The dam's abdomen area was rinsed with 70% EtOH and cut to expose the uterus and embryos. The fetuses were removed and placed in ice-cold Hank’s Balanced Salt Solution (catalog no. 14170-088, Thermo Fisher Scientific, Waltham, MA, USA). The fetuses were rapidly decapitated and brains were removed. Hippocampi were dissected from the brains and placed in ice-cold dissection medium. Hippocampi were incubated in HBSS with 0.25% trypsin (catalog no. 27250-0180, Thermo Fisher Scientific, Waltham, MA, USA) and 0.15 mg/ml deoxyribonuclease (catalog no. DN-25, Sigma-Aldrich, St. Louis, MO, USA) at 37 °C for 15 min. Dissociated hippocampal neurons were plated at a density of 5 × 10^4^ cells per cm^2^ on poly-D-lysine-coated (50 µg/ml, catalog no. P7280, Sigma, St. Louis, MO, USA) multi-wells in G3 medium that contained Neurobasal (catalog no. 21103, Thermo Fisher Scientific, Waltham, MA, USA), B27 (catalog no. 17504, Thermo Fisher Scientific, Waltham, MA, USA), 0.5 mM GlutaMax (catalog no. 35050-038, Thermo Fisher Scientific, Waltham, MA, USA), 25 μM L-glutamate (catalog no. G1626, Sigma-Aldrich, St. Louis, MO, USA), and 10 mg/L gentamicin (catalog no. 15-750-037, Fisher Scientific, Waltham, MA, USA). After 5 days in culture, half of the medium was exchanged for G2 medium (G3 without L-glutamate). Hippocampal neurons were cultured at 37 °C in 95% O_2_ and 5% CO_2_. Cell cultures were inspected under the microscope during the culture. Neurons comprise > 30% of cells at 9th day in vitro (DIV).

### Cell Transfection

Dissociated hippocampal neurons were transfected at 9 DIV with 1.3 μg pEGFP‐N1 (or N1-Sp1-GFP, N1‐E2f3-GFP, or N1‐Ascl1-GFP), 0.2 μg pGL4.10[*luc2*] that encoded the *Ttyh1* gene promoter (or the appropriate pGL4.10[*luc2*] deletion construct), and 0.02 μg pGL4.74[hRluc/TK] using Lipofectamine2000 transfection reagent (catalog no. 11668027, Thermo Fisher Scientific, Waltham, MA, USA) according to the manufacturer’s instructions. As a positive control, cells were transfected with empty pEGFP‐N1, pGL4.10[*luc2*] that encoded the *Ttyh1* gene promoter, and pGL4.74[hRluc/TK]. As a negative control, cells were transfected with empty pEGFP‐N1, N1-Sp1-GFP, pN1‐E2f3-GFP, or N1‐Ascl1-GFP, empty pGL4.10[*luc2*], and pGL4.74[hRluc/TK]. We did not observe transfection of other cell than neurons.

### Cell Lysate Preparation

Cells were washed in 1 × phosphate-buffered saline and lysed in lysis buffer that contained 50 mM KCl, 50 mM PIPES, 10 mM EGTA, 2 mM MgCl_2_, 0.5% Triton X-100, 100 μM phenylmethylsulfonyl fluoride, and 1 mM dithiothreitol, supplemented with protease inhibitors (Roche). The cell lysates were clarified by centrifugation at 12,000 × *g* for 20 min at 4 °C. The protein concentration was determined using Protein Assay Dye Reagent (catalog no. 500-0006, BioRad, Hercules, CA, USA).

### Western Blot

The cell lysates were separated using sodium dodecyl sulfate–polyacrylamide gel electrophoresis and transferred to a nitrocellulose membrane (catalog no. RPN303D, Cytiva, Marlborough, MA, USA). The membrane was blocked with 5% nonfat milk in TBST (0.5 M Tris, 0.9% NaCl, and 0.1% Tween 20, pH 8) for 1 h at room temperature, followed by overnight incubation at 4 °C in TBST that contained rabbit anti-GFP antibody (1:5000 dilution, catalog no. 598, MBL, Woburn, MA, USA), mouse anti-Ttyh1 antibody (1:1000 dilution, catalog no. WH0057348M4, Sigma-Aldrich, St. Louis, MO, USA), or mouse anti-GAPDH antibody (1:1000 dilution, catalog no. MAB374, Millipore, Burlington, MA, USA). After washing with TBST, the membranes were incubated with secondary antibody conjugated to horseradish peroxidase (1:5000 dilution, anti-rabbit horseradish peroxidase-linked antibody, catalog no. AP132P, Sigma-Aldrich, St. Louis, MO, USA; 1:5000 dilution, anti-mouse horseradish peroxidase-linked antibody, catalog no. ab6728, Abcam, Cambridge, MA, USA) in TBST for 2 h at room temperature. The membranes were washed with TBST and developed using Amersham ECL Western Blotting System (catalog no. RPN2108, Cytiva, Marlborough, MA, USA) according to the manufacturer’s instructions. Signal was registered using chemiluminescence western blotting using X-ray film with the automatic film processor. Densitometry was performed with Image Studio Lite Ver 5.2 software. The intensity of Ttyh1 expression was normalized to GAPDH.

### Luciferase Assay

Luciferase activity was measured 24 h after transfection in a luminometer (TD-20/20) using the Dual Luciferase Reporter Assay System (catalog no. E1910, Promega, Madison, WI, USA) according to the manufacturer’s instructions. Briefly, cells were lysed with Passive Lysis Buffer, incubated at room temperature for 10 min on an orbital shaker, and centrifuged at 10,000 × *g* for 10 min. Luciferase Assay Reagent II and Stop&Glo reagent were sequentially added to the cell lysate to measure the activity of firefly (*Photinus pyralis*) and *Renilla* (*Renilla reniformis*), respectively. The activity of firefly was normalized to the activity of *Renilla*. Data were collected from four biological replicates. Three technical replicates were performed for each sample.

### Statistical Analysis

Data were collected from four independent experiments. Three technical replicates were performed for each sample. The results are presented as mean ± standard error of the mean (SEM). The statistical analysis was performed using Prism 5.0 software (GraphPad, La Jolla, CA, USA). Datasets were tested using the two-tailed Student’s *t*-test. Values of *p* < 0.05 were considered statistically significant.

## Data Availability

The manuscript has no associated data.
